# Machine learning approach towards explaining water quality dynamics in an urbanised river

**DOI:** 10.1038/s41598-022-16342-9

**Published:** 2022-07-19

**Authors:** Benjamin Schäfer, Christian Beck, Hefin Rhys, Helena Soteriou, Paul Jennings, Allen Beechey, Catherine M. Heppell

**Affiliations:** 1 Queen Mary University of London, School of Mathematical Sciences, Mile End Road, London, E1 4NS UK; 2 Faculty of Science and Technology, Norwegian University of Life Sciences, 1432 Ås, Norway; 3 Institute for Automation and Applied Informatics, Karlsruhe Institute of Technology, 76344 Eggenstein-Leopoldshafen, Germany; 4 The Alan Turing Institute, 96 Euston Road, London, NW1 2DB UK; 5 The Francis Crick Institute, Flow Cytometry Science Technology Platform, London, UK; 6 Thames Water, Clearwater Court, Vastern Road, Reading, RG1 8DB UK; 7 River Chess Association, Croxley Green, UK; 8 Chilterns Chalk Streams Project, Chilterns Conservation Board, Chinnor, Oxfordshire OX39 4HA UK; 9 Queen Mary University of London, School of Geography, Mile End Road, London, E1 4NS UK

**Keywords:** Hydrology, Applied mathematics, Scientific data, Computer science, Environmental impact

## Abstract

Human activities alter river water quality and quantity, with consequences for the ecosystems of urbanised rivers. Quantifying the role of human-induced drivers in controlling spatio-temporal patterns in water quality is critical to develop successful strategies for improving the ecological health of urban rivers. Here, we analyse high-frequency electrical conductivity and temperature data collected from the River Chess in South-East England during a Citizen Science project. Utilizing machine learning, we find that boosted trees outperform GAM and accurately describe water quality dynamics with less than 1% error. SHapley Additive exPlanations reveal the importance of and the (inter)dependencies between the individual variables, such as river level and Wastewater Treatment Works (WWTW) outflow. WWTW outflows give rise to diurnal variations in electrical conductivity, which are detectable throughout the year, and to an increase in average water temperature of 1 $\rm{^o}C$ in a 2 km reach downstream of the wastewater treatment works during low flows. Overall, we showcase how high-frequency water quality measurements initiated by a Citizen Science project, together with machine learning techniques, can help untangle key drivers of water quality dynamics in an urbanised chalk stream.

## Introduction

Across the globe human activities, such as urbanisation, are causing changes to catchment water cycles that have profound impacts on the water quantity, quality and the ecology of rivers^1,2^. Urbanisation changes the hydrology of a catchment in multiple ways^3^. The introduction of impermeable surfaces, together with artificial drainage systems, can increase peak flows in rivers^3^, reduce hydrological response times to rainfall^4,5^ and reduce baseflow and groundwater recharge^6^. In many countries combined sewer systems carry domestic and industrial wastewater to wastewater treatment plants under dry conditions, along with stormwater drainage from paved areas when it rains. Treated effluent discharge can cause distinctive flow patterns in rivers dictated by human activity^7,8^.

The human activities associated with urbanisation also have impacts on chemical water quality, with lots of effort focused on characterising water quality changes in urban rivers during storm events^9–11^, and the ‘first flush’ phenomenon^12,13^. Combined sewer overflows can also significantly impact water quality following intense rainfall events when the capacity of wastewater treatment works has been exceeded^14–17^. We also know that urban streams tend to have higher mean electrical conductivity and major ion concentrations in comparison to their rural counterparts^18–20^, which arises from a combination of point and diffuse pollution sources. For example, chloride, sulphate, sodium and potassium are common electrolytes in urine and therefore concentrated in wastewater^19^. Determining the main sources of individual ions in urban systems, however, has proven to be challenging^21^. Such elevated solute levels are now leading ecologists to hypothesise about potential implications of elevated ionic concentrations for the health and resilience of urban stream ecosystems^22^.

Human activities are causing widespread degradation of water quality in rivers with consequences for ecological health^23^. These activities lead to changes to the water quality of receiving waters which operate over nested timescales from hours (in response to rainfall events) to daily, seasonal and inter-annual cycles^24,25^. To fully understand the impact of human activities on rivers we need to tease apart human-induced and natural variations in water quality. To do this we need access to high-resolution and long-term monitoring data of urbanised systems such as becomes available from using real-time high-frequency water quality sensors^26^.

With detailed data available, machine learning (ML) is becoming an important alternative to process-based or traditional statistical models. This development is further accelerated whenever ML models show superior predictive performance^27^ when compared to traditional approaches. For example^28^, recently demonstrated that machine learning can be used to detect untreated wastewater discharges when trained with 15-mins flow data from wastewater treatment works (WWTW). Specific machine learning techniques include Generalized Additive Models (GAM) and boosted trees. GAM techniques have been used to investigate correlations between chlorophyll *a* and other water quality parameters^29^. Meanwhile, boosted tree analysis has been used, for example, to rank the importance of factors affecting nitrate concentration in groundwater, and to create nitrate vulnerability maps^30^. The underlying idea of boosted trees is to combine many “weak learners”, namely simple regression trees, into one ensemble predictor^31^. Boosted trees often outperform neural networks, in particular on tabular data^32^, but they do have problems predicting future events and extrapolating beyond previously recorded values.

When applying machine learning, it is important to avoid black-box solutions, as these do not provide process-based scientific insight^33^. With the advent of ’eXplainable’ or ’Interpretable’ machine learning (IML), machine learning has been enhanced to highlight understanding of relevant relationships contained in the data. However, these IML methods have so far not been used widely for water quality analysis^27,34,35^. Here we show how IML can be used to determine the relative importance of different environmental and human factors controlling water quality dynamics, and to tease out the nature of the relationships between river level and electrical conductivity or temperature. In this case, we use boosted trees and interpret these traditional black-boxes via Shapley values^36,37^, comparing their overall performance with a more traditional GAM approach. We stress that our analysis in itself is transparent and reproducible: We make our code available online and all our results are based on publicly available and open source packages, e.g. in Python and R.

In the UK, one of the river types seemingly under the most pressure from human activity are the groundwater-fed chalk streams. Most of the world’s chalk streams (224 rivers) are located in England^38^, and they are considered of international importance for their characteristic hydrology, water quality, ecology and aesthetics. However, 77% of these rivers fail to meet ‘good’ ecological status as defined by the European Union (EU) Water Framework Directive^39^, with pressures arising from over-abstraction, farming activities and urbanisation. Despite being designated under Annex 1 of the Habitats Directive they are more likely to be in a ‘poor’ or ‘bad’ status than the average river in England and Wales^38^. Chalk streams on the dip slope of the Chilterns Area of Outstanding Natural Beauty (AONB) typify the issues facing many UK rivers which are located in rapidly urbanising areas, where treated effluent comprises a high proportion of the total river flow. Furthermore, climate change threatens the resilience of these river ecosystems^40^. Under climate change scenarios of hotter, drier summers the proportion of treated effluent to groundwater in these systems may rise further. In addition, predicted increases in the frequency of intense rainfall may put additional pressure on sewage treatments works that receive water from combined sewer networks, leading to more frequent storm tank discharge events, further modifying water quality. Such possibilities provide additional impetus for us to develop means of understanding the significance of different (natural and human) contributions to water quality patterns in urbanised rivers. Among these contributions, wastewater management is among the most pressing issues for water quality^41,42^, in particular during low-flow situations.

Finally, our research also relates to Citizen Science endeavours which are gaining prominence and interest in academic literature due to opportunities to collect datasets that may not have been possible without local and public support. These large data sets are critical to enable any machine learning application. Furthermore, citizen scientists promote the subject in the local community and thereby increase awareness, in particular for environmental issues^43,44^.

Here, we focus on the River Chess, which is a pilot catchment for ‘The Smarter Water Catchment Initiative’ created by Thames Water, which aims to improve catchment management through partnership projects that tackle multiple challenges and embrace Citizen Science. The initiative has offered us the opportunity to collect a long-term time series of temperature and electrical conductivity at fifteen-minute intervals using sensors maintained by Citizen Scientists. In this paper the focus is on variations in time series of temperature and electrical conductivity (as a proxy for total dissolved solutes) because these are water quality parameters that can be altered by anthropogenic drivers with important effects on other critical ecological processes such as metabolism^26,45^ and because they are cheap and easy for Citizen Science groups to monitor.

Our overall aim is to demonstrate the use of machine learning tools, in particular GAM, boosted tree and SHAP analysis, to analyse the spatio-temporal patterns in temperature and electrical conductivity arising from point and diffuse urban runoff in a groundwater-fed river. We use these machine-learning tools to tease out the influence of a wastewater treatment works on the observed spatio-temporal patterns. Finally, in light of our findings we assess the ways in which electrical conductivity datasets such as ours might prove useful for Citizen Science groups exploring water quality issues in urbanised rivers.

## Results

### Data overview and statistical analysis

**Figure 1:**
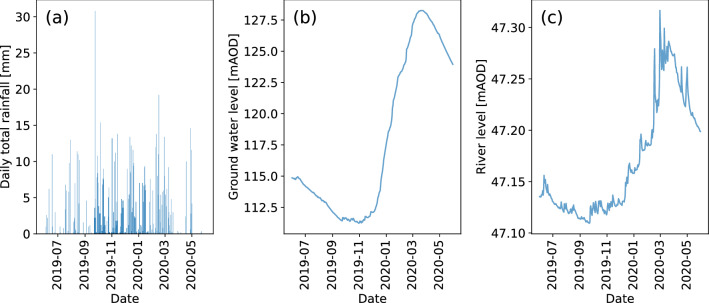
Times series graphs of (**a**) Daily total rainfall (Chenies, EA station); (**b**) Groundwater level (Ashley Green, EA station); and (**c**) River level of River Chess at Rickmansworth from 1st June 2019 to 1st June 2020 (EA Gauging station)^46^.

Our monitoring time period covers a period of drought with exceptionally low flows (September 2019) and low groundwater levels in the catchment; increasing to exceptionally high flows in February / March 2020 as groundwater levels rose in response to high autumn and winter total rainfall in 2020, accompanied by high-intensity rainfall events. The groundwater and river levels display a clear seasonal cycle in response to the changing rainfall patterns in the catchment (Fig. 1).

**Figure 2:**
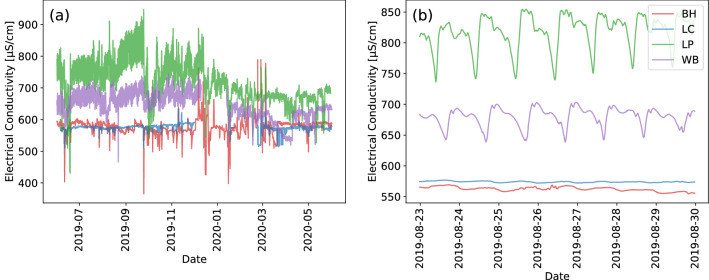
Electrical conductivity time series (**a**) June 2019 to 2020; and (**b**) 23 August to 30 August 2019.

**Figure 3:**
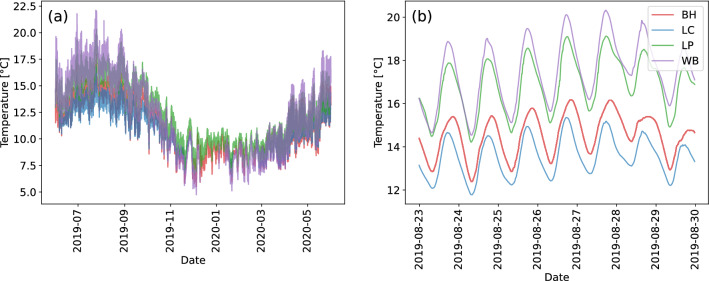
Temperature time series (**a**) June 2019 to 2020; and (**b**) 23 August to 30 August 2019.

Seasonal dynamics are also observed in the electrical conductivity dataset at the sites downstream of Chesham WWTW (LP and WB), see also Methods for a map. At these sites, electrical conductivity is highest whilst groundwater and river levels are low, and decreases once groundwater and river levels rise (Fig. 2a), whereas there is no obvious seasonal pattern in electrical conductivity at the sites upstream of the WWTW (BH and LC). River water temperature displays a strong seasonal signal at all sites, with higher water temperatures in the summer (July to September 2019) declining over the autumn to a winter low, and then rising again in the Spring (Fig. 3a). If we plot one week of electrical conductivity data (Fig. 2b) we can also observe daily cycles in electrical conductivity downstream of the WWTW which are not observable at the upstream locations (Fig. 2b). River water temperature also exhibits marked daily variation at all sites (Fig. 3b).

**Figure 4:**
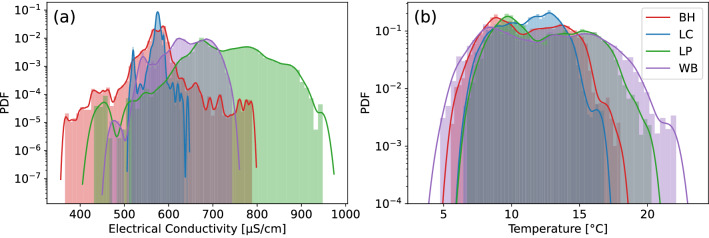
Normalised histograms of (**a**) electrical conductivity; and (**b**) temperature for all sensor locations. Note the log-scale on the y-axis, which highlights the heavy tails in BH.

The time series can be more systematically analysed via its PDF (extracted from histograms), to show overall differences between sites (Fig. 4), with important statistical parameters summarised in Table 1. Mean electrical conductivity is highest at the location 2 km downstream of the WWTW (734 $\rm{\mu S cm^{-1}}$ at LP) and is lower 5 km further downstream (648 $\rm{\mu S cm^{-1}}$ at WB). This contrasts with a lower electrical conductivity upstream of the WWTW (565 and 575 $\rm{\mu S cm^{-1}}$ at LC and BH respectively). The electrical conductivity datasets do not follow Gaussian distributions, and instead display heavy tails, especially upstream of the WWTW where the kurtosis $\kappa >3=\kappa _\text {Gaussian}$^47^. In contrast, the river water temperature shows a gradual increase in mean values with increasing distance downstream from Chesham, with a $1.1^{\rm{\circ } C}$ difference between BH and WB, and kurtosis $\kappa < 3$.

**Table 1:** Mean $\mu$, standard deviation $\sigma$ and kurtosis $\kappa$ of electrical conductivity and temperature at all four measurement sites.

	Electrical conductivity [$\rm{\mu S/cm}$]			Temperature [$\rm{{^\circ }C}$]		
	Mean $\mu$	Stan. Dev. $\sigma$	Kurtosis $\kappa$	Mean $\mu$	Stan. Dev. $\sigma$	Kurtosis $\kappa$
BH	575.2	25.58	16.82	11.2	2.43	2.0
LC	574.49	10.59	17.74	11.33	1.96	2.21
LP	733.57	67.1	3.02	12.31	2.81	2.0
WB	647.86	43.2	2.92	12.34	3.61	1.97

Note that the measurement period includes Spring 2020, i.e. the onset of the Covid-19 pandemic in Britain. Analysing the data we find small but not significant impacts from the March lockdown in the UK, see code for details.

### Evaluating critical periodicity in electrical conductivity and temperature using Fourier analysis

**Figure 5:**
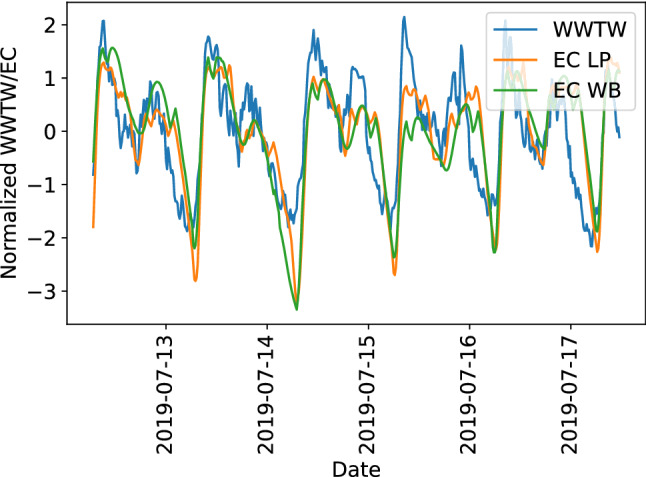
Normalized time series of wastewater treatment works (WWTW) treated effluent discharge and electrical conductivity (EC). EC was measured at LP and WB and shifted by 2.5 and 8.5 hours respectively to account for the time delay of the sewage treatment discharge to reach the sensor site.

We observe a clear alignment between the discharge of treated effluent from the wastewater treatment works (WWTW) and electrical conductivity of river water (Fig. 5). To account for the time delay between the WWTW discharge being recorded at the plant and the water reaching our downstream sensors at the LP and WB sites, we shift the river water electrical conductivity measurements back by about 2.5 and 8.5 hours respectively.

**Figure 6:**
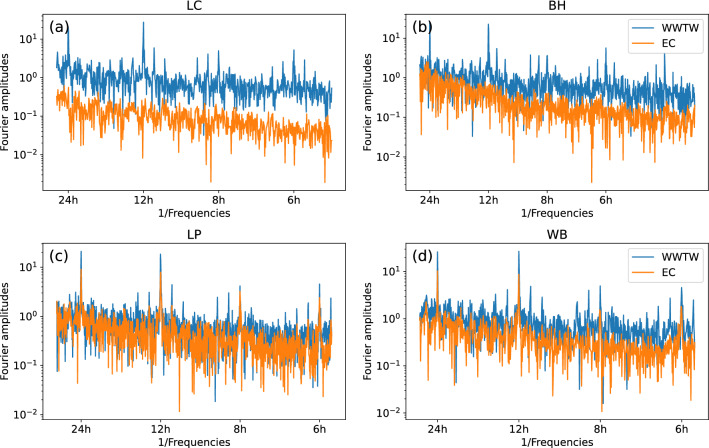
Fourier analysis of wastewater treatment works treated effluent discharge and EC. We plot the Fourier amplitudes of both the electrical conductivity (EC) and the wastewater treatment works (WWTW) discharge at all sites. We note relevant frequency peaks at 24, 12, 8 and 6 hours in the WWTW discharge as well as in conductivity for LP and WB.

To further quantify the relationship between treated effluent discharge from the WWTW and EC, we perform a Fourier transform analysis to expose the main frequencies present in the system (Fig. 6). We compare the Fourier analyses of the treated effluent discharge with the Fourier analysis of the river water EC at all four measurement sites. Notably, both WWTW discharge and electrical conductivity at the two downstream locations (LP and WB) show pronounced peaks at frequencies of 24, 12, 8 and 6 hours, while we do not observe any such cyclic behaviour at LC or BH.

### Data-driven modelling of EC dynamics

Next, we pursue two data-driven approaches to describe the electrical conductivity (EC) as a target variable (*y*) characterized by $p=7$ features: Two local variables: Temperature, pH (local sensor variables) and five global variables: rainfall, river level and time stamps, split into month, day and hour. For all time series we use data from 1st June 2019 to 1st June 2020, removing NaN entries where necessary, then we perform both a GAM and boosted tree analysis, see also Methods. Note that the river level at all sites uses the values recorded at Rickmansworth, which is several kilometers downstream from all measurement sites. We later also consider a model run where we align the local time series of a sensor with the station in Rickmansworth.

#### GAM for EC

**Figure 7:**
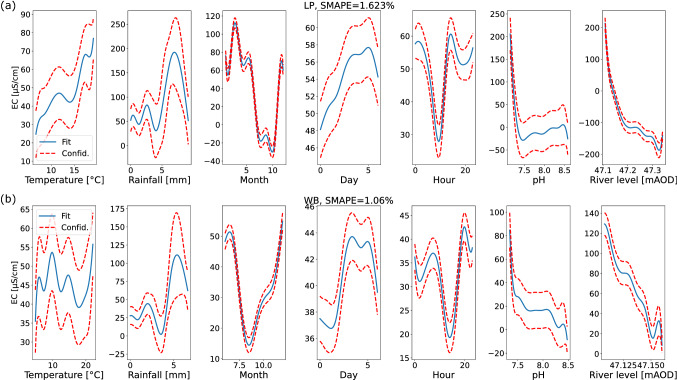
Results of the GAM analysis for LP (**a**) and WB (**b**). Best fitting splines of the different features $x_{i}$ and their influence on the electrical conductivity (EC) in the fully-fitted GAM approach. The blue curve gives the best fit and the red dashed lines envelop a single confidence interval ($68\%$ assuming an underlying Gaussian uncertainty). Finally, we report the symmetric mean absolute percentage error (SMAPE)^48^ when the model is applied to the previously withhold test set.

We performed a GAM (generalized additive model) analysis on all sites but focus here on the two downstream locations, while the results for the two upstream locations are provided in the Supplement. The most important features, based on the magnitude of their contributing splines, are the pH value and river levels, both being negatively correlated with electrical conductivity (Fig. 7). The deviation between model and test set is $\text {SMAPE}\approx 1...2\%$, where SMAPE stands for the symmetric mean absolute percentage error^48^.

#### Boosted tree analysis of EC

**Figure 8:**
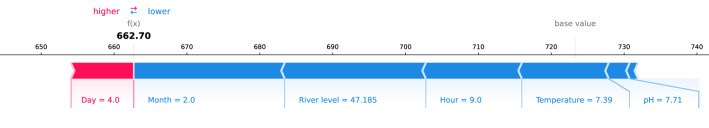
Explanation of boosted tree results via SHAP. Starting from a base value (here approx. 723), each feature pushes the prediction for the electrical conductivity value to lower (blue) or higher (red) values relative to the base value (ensemble average). Here, we explain one EC measurement at the LP measurement site via SHAP.

We then apply a boosted tree approach, using SHAP to interpret the results. First, let us discuss how an individual explanation is obtained (Fig. 8): The “base value” (mean conductivity for LP) of about 723 $\rm{\mu S/cm}$ is altered in this specific data point by the positive impact of the “day” feature (which day in the week), while “month”, “river level”, “temperature” and “hour” all push the prediction to a lower value. Hence, the model predicts a value of 662.7 $\rm{\mu S/cm}$, with the most influential feature being the month.

**Figure 9:**
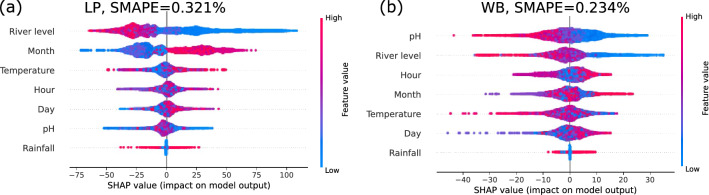
Feature ranking of the boosted tree via SHAP. The features are sorted by their importance in predicting deviations from the mean EC for LP (**a**) and WB (**b**), see also Fig. 8. As in the GAM approach, we report the symmetric mean absolute percentage error (SMAPE) of the model when applied to the test set.

We continue with a more systematic study, by ranking the impact of each feature on the prediction, thereby moving from a single local explanation to global model properties^37^. Here, river level, temperature, pH and the time (month or hour) are among the most important descriptive features (Fig. 9). As before, negative SHAP values push the electrical conductivity prediction towards lower amounts, while positive values push the prediction to higher ECs indicative of greater total dissolved solute values. The colours indicate the feature value, going from high (red) to low (blue). Thereby, we obtain a first impression on the dependencies here: The river level feature is mostly red for negative values and blue for positive SHAP values, i.e. it is negatively correlated with the conductivity. Finally, we compute the deviation between model and test set as $\text {SMAPE}\approx 0.2...0.4\%$.

**Figure 10:**
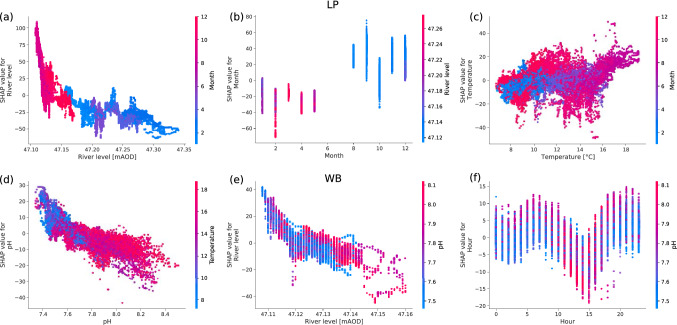
Partial dependency plots of the EC boosted tree for LP (**a-c**) and WB (**d-f**). We plot the three most important features of both downstream measurement sites. The colours (and left-hand side axis) highlight second-order interactions between the plotted feature and a secondary feature.

To investigate how each feature contributes to the model, we analyse partial dependency plots of the three most important features; river level, month and water temperature for LP and river level, pH and hour of day at WB (Fig. 10). In each partial dependency plot, the color displays the values of the interacting feature that explains most of the observed variance (in (Fig. 10a the month). We consistently observe a negative relationship between electrical conductivity and the river level (Fig. 10a,e), as well as electrical conductivity and the pH value (Fig. 10d), i.e. higher river level or pH value lead to a lower EC prediction. Note that all plots use colour-code to display a secondary feature that explains most variances of the primary feature prediction. So we can observe that low river levels in months 8 through 12 (August to December) are associated with highest river water electrical conductivity at LP (Fig. 10a). At WB the highest electrical conductivity values in river water are associated with low pH of 7.4 to 7.8 during months 8 through 12 (August to December). Furthermore, there are interesting temporal trends in the hour and month features. For example, at WB the afternoons (13:00 to 16:00) were characterised by lower electrical conductivity in river water at times when pH tended to be higher (7.8 to 8.1). Feature ranking and partial dependency plots for upstream sites are displayed in the Supplement.

**Figure 11:**
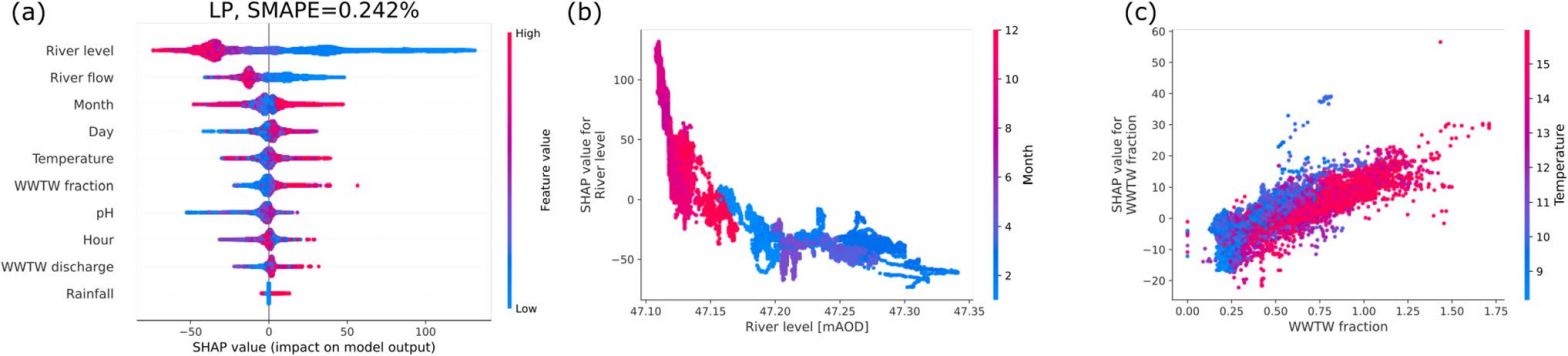
Feature ranking and partial dependency plots of the extended EC model at the LP site. We plot the feature ranking (**a**), the most important feature (**b**) and the dependency on the fraction of flow from the WWTW (**c**). Note that surface water-groundwater exchange in the river between the WWTW and gauging station site can lead to a value for WWTW fraction $> 1$.

Extending the previously derived results, we incorporate three new features: The total river flow, the total WWTW discharge and the WWTW fraction (ratio of WWTW discharge and the total flow), adjusting for the time delay between the different measurement stations (see Supplements for details). We highlight that incorporating such additional useful features improves the performance of the model, see Fig. 11. Notably, the most important features, river level and month still remain important in this extended feature set. Furthermore, the partial dependency (Fig.  11b) for the river level remains almost unaltered by adding a new feature. Finally, note that the EC value within the model is almost linearly dependent on the fraction of flow attributed to the WWTW (Fig. 11c), very much in line with alignment in the Fourier spectrum and time series observed earlier: Higher WWTW discharge indicates a higher EC. This linear dependency is much clearer in the model compared to a simple scatter plot.

#### Boosted tree analysis of temperature

**Figure 12:**
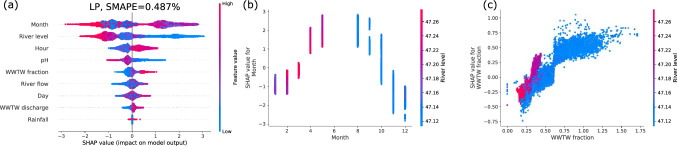
Temperature analysis of the LP site. We plot the feature ranking (**a**), the most important feature (**b**) and the dependency on the fraction of flow from the WWTW (c).

Having analysed EC in detail, we investigate temperature dependence next. Using the extended data set (i.e. including absolute flows and the WWTW fraction) we achieve very good fits, see Fig. 12: $\text {SMAPE}\approx 0.5\%$ deviation on average. As in the EC analysis, the total river level is ranked very highly in our feature list but the month is ranked even higher, pointing to the strong seasonal dependency of the temperature. While the WWTW fraction is not ranked among the top three features here, slight variations of the hyperparameters lead to a reordering of the feature ranks (see Code for details) and the dependency of temperature on the WWTW fraction feature remains robust. An increase in the WWTW fraction is accompanied by an increase in temperature. In the model shown here, elevated WWTW discharge can influence the temperature prediction by up to $1^{\rm{\circ }}C$ when river levels are at their lowest (blue data points, Fig. 12c). When river levels are high (red to purple data points, Fig. 12c) the WWTW fraction is low and there is less of an effect on temperature.

## Discussion

Contributions of flow to the river from the WWTW at Chesham fluctuate from 40 to 70$\%$ depending on groundwater levels, and on the short-term impacts of rainfall events. This important point source contribution to river flow also gives rise to significantly higher mean electrical conductivity in surface water downstream of the WWTW outfall (Fig. 4a, Table 1). Thus, this study provides further evidence of the role that treated WWTW inputs play in causing elevated electrical conductivity in urbanised river reaches^18–21^. Although the WWTW outfall is a point source of solutes to the river system, the WWTW receives water from a combined sewer system comprising household and industrial effluent along with road runoff from diffuse sources, as is common in England. Therefore, the composition of solutes in the treated effluent from the WWTW will vary depending on human activities as well as rainfall. In addition, the contributing sewer network is subject to groundwater ingress during periods of elevated groundwater levels (e.g. March 2020 onwards) adding to the list of factors controlling the electrical conductivity of the effluent. Nevertheless, despite these potential causes of variations in electrical conductivity in the WWTW effluent (this determinand is not monitored by the water company), we do see consistent temporal trends in riverine electrical conductivity arising from this point source contribution to the river.

### The role of the WWTW in controlling seasonal and daily variations in riverine electrical conductivity and temperature

In a natural river system baseflow is considered relatively high in EC because groundwater is rich in solutes compared to the quickflow components of the hydrograph, which are dominated by the rainfall and shallow subsurface flows^49^. In this groundwater-fed system the seasonal and supra-annual variations in river level and discharge are controlled mainly by groundwater level (Fig. 1). We observe that EC remains stable throughout the year in our groundwater spring site (LC) upstream of the WWTW, indicating little variation in EC arising from changing depths or sources of groundwater (Fig. 2a, Table 1). Our upstream urban site (BH) has a more variable EC signal. This variability arises from rainwater inputs to the channel from rapid urban runoff, which causes sharp drops in EC in response to high intensity rainfall events (Fig. 2a, Table  1). The greatest variations in EC, however, are observed at the sites downstream of the WWTW. At these sites (LP and WB) the influence of river level and the fraction of WWTW effluent on seasonal cycles of EC are clear, with highest EC recorded when river levels are at their lowest (Fig. 10), and the contribution of WWTW effluent to baseflow is highest (Fig. 11b,c).

Not only are there marked seasonal changes in EC, but also marked patterns on 24 hour, and shorter timescales caused by changes in treated effluent discharge from the WWTW (Figs. 5, 6c,d, 10f). The WWTW has two periods of peak effluent discharge; at 13:00 to 14:00 GMT and 21:00 to 22:00 GMT associated with patterns in human domestic activities in the catchment. This electrical conductivity signature from these peaks in activity is attenuated downstream, but remains observable 5 km downstream of the outfall; and even when river levels are high during Spring (Feb to May 2020). This ‘signature’ can be used to estimate an average water travel time or velocity between sites. The approximate travel time of water from the WWTP to LP and WB is 2.5 and 8.5 hours respectively. Therefore, daytime (09:00 to 17:00) conditions in the river at WB correspond to a period of lowest daily contributions from the WWTW.

Here we consider whether daily changes in electrical conductivity might be measurable on other urbanised rivers in the UK and how transferable our observations might be? In this regard a useful parameter to consider is ‘dilution factor’, which is defined as the ratio of river flow at the catchment outlet to total domestic wastewater effluent^50^. Dilution Factor is used in ecological risk assessments under EU legislation to predict a ’worst case exposure’ of surface water to chemicals from treated effluent^51^. For these analyses a fixed dilution factor of ten is used. On the basis of treated effluent flow data from Chesham, and the river flow at Rickmansworth gauging station we estimate a mean dilution factor of 2.67 during our study, with a minimum 0.87 during the drought period at the beginning of our study, and maximum of 6.81 in February/March 2020. Throughout this entire period diurnal cycles of electrical conductivity were observable in the river.^50^ estimate that 25$\%$ of UK rivers are estimated to have a mean dilution factor of $< 6.26$, and these rivers are mainly found in highly populated regions such as South East England. On this basis, we predict that diurnal changes in electrical conductivity could be observable in many lowland rivers in the UK, although these cycles may be complicated by the effect of more than one wastewater treatment works upstream of a river measurement station. There is no comprehensive review of dilution factors in European rivers, but recent analysis of large German WWTWs of size $> 10,000$ population equivalents has suggested that 60$\%$ of dilutions factors fall below 10 suggesting that diurnal changes in electrical conductivity might be more widely observable^52^.

The partial dependency plots from the boosted tree analysis also allows us to consider the relationship between the proportion of flow from the WWTW (using electrical conductivity as our proxy) and pH at WB. Figure 10e shows that the periods of highest contributions from the WWTW to overall flow in the river are associated with lowest pH conditions. Figure 10f indicates that the daytime conditions in the river at WB (09:00-17:00), when the contribution of flow from the WWTW is lowest, are associated with the highest pH conditions. During daylight hours photosynthesis will also give rise to more alkaline river water, but this analysis hints that a combination of photosynthesis and treated effluent might be increasing the amplitude of pH cycling in the river.

Finally, the analysis also enables us to unravel the features that control river water temperature. The SHAP analyses confirms the previously known important interplay between season and river level on temperature^53^. For example,^54^ have previously used air temperature and water level to predict hourly changes in water temperature using an artificial neural network modelling approach. Our modelling also shows that the fraction of flow from the upstream WWTW is positively correlated with a change in river water temperature of ca. 1$\rm{^{o}C}$ during periods of low river levels warranting further research in this area. Especially given that summer water temperatures during low flow conditions exceeded 20 $\rm{^{o}C}$; temperatures which can affect the recruitment success of salmonids such as brown trout, *Salmo trutta*, and European grayling, *Thymallus thymallus*^55^. Wastewater treatment works are a source of warm water that has received little attention to date, but a UK-national-scale analysis by^56^ have shown that there is potential to recover heat from wastewater treatment works to help meet climate change targets, with the additional benefit of reducing environmental impacts on rivers. Our analysis indicates that a mitigation option such as heat recovery from treated effluent discharge may benefit chalk streams, such as the River Chess, which receive high proportions of their flow from treated effluent. This is especially important at a time when population growth is predicted to increase the volume of treated effluent reaching the river on a daily basis.

### Use of machine learning for interpreting trends and factors controlling electrical conductivity

Both the GAM and SHAP analyses showed good agreement with regards to the influence of the different variables on electrical conductivity. For example, pH and river level were shown by both techniques to be negatively correlated with electrical conductivity. However, the SHAP approach achieved almost a one order of magnitude improvement in model performance in comparison to the GAM as measured by symmetric mean absolute percentage error ($\text {SMAPE} =0.2$ and 1.5 respectively). Critically the boosted tree analysis is easy to visualise and interpret using the SHAP analysis, and the regression tree approach therein enables variable interactions to be considered, whilst the GAM approach assumes independent variables. In addition, the boosted tree approach deals with non-linearity and can perform well on tabular data^31^. An alternative approach might be neural networks but these typically do not allow for such a good interpretation as the SHAP approach illustrated here^57^.

Here, we utilized Python packages and in particular the idea of automated machine learning^58^, hopefully making these machine learning techniques easier to try for a broad research community. While analysis via boosted trees is also available in R^59^, e.g. via the caret package^60^, a detailed interpretation of the derived model, as done here via SHAP values, is not yet available therein, but could be included in the future.

### Use of high-frequency electrical conductivity sensors for Citizen Scientist groups

‘Roving’ low cost electrical conductivity sensors can be moved around a catchment to help investigate spatial patterns in contaminants. These sensors could be used to help identify potential sources of pollution due to sewer mis-connections, and embedded as a tool in ‘Outfall Safaris’ (https://catchmentbasedapproach.org/learn/outfall-safari-guide/) to identify locations characterised by high total dissolved solutes worthy of further investigation. Measurements of electrical conductivity could be combined with dissolved oxygen to link measurements of dilution capacity to ecological function, and ecological status under the Water Framework Directive.

Here we also show the advantages of high-frequency monitoring of electrical conductivity for rivers groups. High-frequency monitoring of water quality determinands is an increasingly common practice for regulators and scientists in urbanised catchments^20^,^49^ yielding important insights into the causes of temporal variations in water quality^61^. Recent advances in sensor technologies using the Internet-of-Things (IoT) approaches^62^ is making high frequency monitoring of electrical conductivity and temperature a potential cost-effective investigative tool for Citizen Science groups and participatory research.

Understanding diurnal cycles in water quality should be important to rivers groups. The cyclical daily variation in electrical conductivity data could be used as basis for planning urban water quality monitoring campaigns. Such information could dictate when to focus sampling activity and effort over a diurnal cycle to examine the likely best and worst-case scenarios with respect to chemical concentrations arising from point source inputs (e.g. nutrients and pharmaceuticals). Understanding how the electrical conductivity signal attenuates downstream would also enable Citizen Scientists to identify the optimum sampling times at different points downstream of a point source input. These types of analyses could be embedded into toolkits currently being developed by initiatives such as CaBa in the UK to help groups prioritise action plans for their rivers in conjunction with water companies and regulators.

### Magnitude of variation in electrical conductivity in relation to ecosystem health

Human activities are increasing concentrations of total dissolved solutes in freshwaters globally. Although much of the focus is currently on human health effects (e.g. groundwater thresholds of 1880 $\rm{\mu S cm^{-1}}$ for drinking water protected areas designated under the Water Framework Directive) and irrigation, potential ecological effects are now receiving more attention^22^, along with recommendations for developing ecological criteria for specific ions and their mixtures. For example, future good practice may involve reduction of salt loads though minimising point source discharge of salts to freshwaters through resource extraction^22^. The observed changes in electrical conductivity in the River Chess although unlikely to pose an ecological risk per se will be indicative of the changing loading of effluent-derived chemicals to the river^63^. In-situ sensors do not currently exist to continuously measure these emerging chemicals at high frequency and electrical conductivity could therefore be considered as a proxy when developing risk criteria for urbanised rivers^49^.

Changes in electrical conductivity also arise, in part, due to variations in concentrations of anions such as chloride^61^, phosphate and nitrate in the river, and suggest that a more detailed investigation into the high-frequency variations in these anions is warranted in order to improve accuracy of calculations of loading. Understanding how these diurnal cycles of these chemicals vary with seasonal changes in discharge is also critical to understanding the potential overall influence of a particular point source discharge on the biological function of the river system. Although the impact of treated effluent on stream function has shown to be marked in semi-arid and Mediterranean regions^64–66^ our data demonstrate that limited dilution capacity could also mean that treated effluent has a critical influence on in-stream biogeochemical cycling in temperate, urbanised streams.

## Conclusions

SHAP analyses - a method from the domain of interpretable machine learning (IML) - has opened up a black box model to provide useful insights into inter-dependent factors controlling cycles of electrical conductivity and temperature in an urbanised river. These analyses have enabled us to demonstrate that the fraction of WWTW effluent making up total streamflow is a critical variable aligning with seasonal and diurnal cycles of electrical conductivity and temperature in this urbanised chalk stream. As the dilution factors associated with treated effluent in the River Chess are comparable to many other rivers in England and Wales, we hypothesise that cheap, high-frequency measurements of electrical conductivity could help explore the influence of WWTWs in other urbanised river systems. We have also used this analysis to demonstrate the influence of a WWTW on river water temperature, highlighting that in this case the WWTW is associated with a 1 $\rm{^oC}$ increase in water temperature, at a distance of 2km from the treated effluent outlet during the lowest flow conditions. Furthermore, other Citizen Science groups could use cheap and cost-effective electrical conductivity measurements to direct water sampling activities in urban rivers. Using simultaneous upstream and downstream measurements of electrical conductivity they may be able to target optimal times to measure different water quality and ecological parameters, and quantify travel times of water through urban river systems.

## Methods

### Site selection

**Figure 13:**
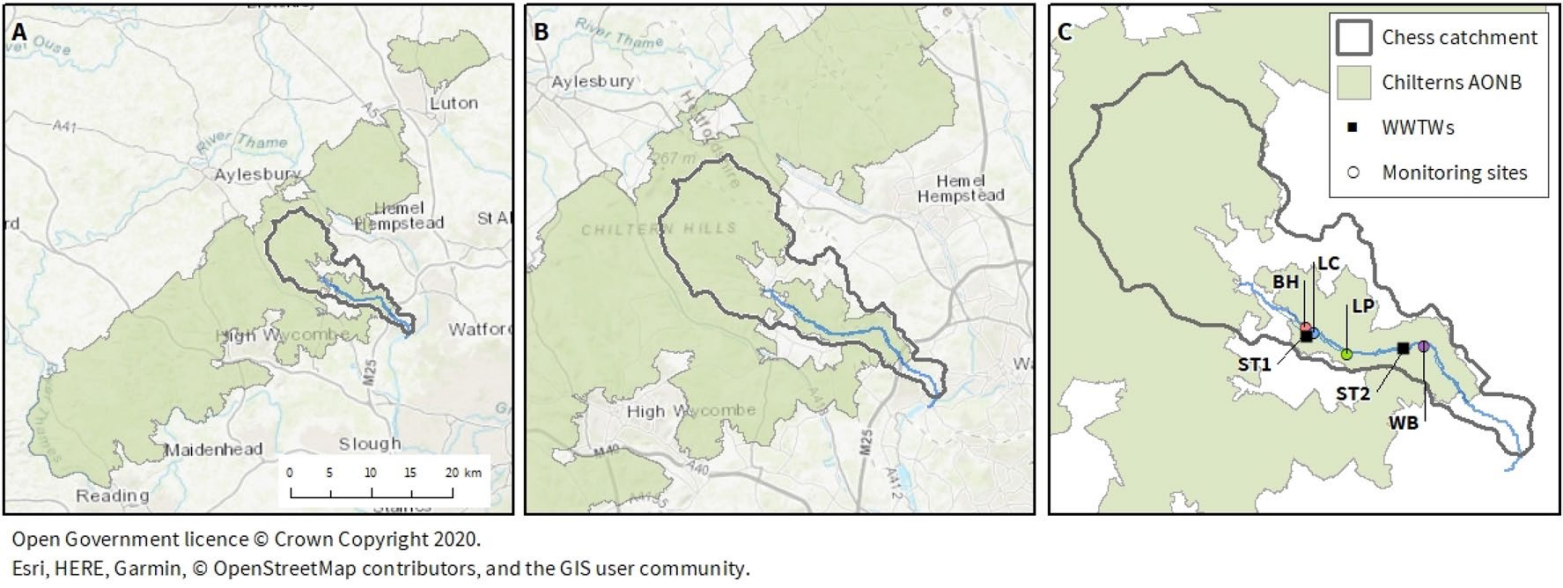
Map showing location of (**a**) Chiltern Area of Outstanding Natural Beauty (AONB) in South East England (**b**) River Chess flowing from the Chilterns AONB; and (**c**) the four monitoring sites in the River Chess: BH, LC, LP and WB. ST1 and ST2 indicate the location of the two Wastewater Treatment Works on the river. Maps created using open data from OpenStreetMap available under the Open Database License, see^67^ for details.

The River Chess (8 miles in length, catchment area 105 $\rm{km^2}$) is one of nine low gradient chalk streams draining the dip-slope of the Chilterns Area of Outstanding Natural Beauty (AONB), see Fig. 13 for a map. The standardised average annual rainfall for the catchment is 753 mm (1961–1990,^46^), and the baseflow index is 0.95. Land cover in the catchment is mixed with 12% urbanised, 18% woodland, 35% grassland and 35% arable land cover. The winterbourne (ephemeral) sections of the river around the town of Chesham are urbanised and channelised with numerous artesian wells in addition to groundwater springs. Downstream of Chesham the landscape becomes more rural, and grassland and arable land use dominate until the river reaches its confluence with the River Colne at Rickmansworth. Mean annual flow at the Rickmansworth gauging station is 0.54 $\rm{m^3s^{-1}}$^46^. Treated effluent from Chesham Wastewater Treatment Works (population equivalent = 37,300; ST1 in Fig. 13) comprises around 40 to 70% of the flow in the river downstream of Chesham depending on the flow conditions. ST2 in Fig. 13 is a small rural wastewater treatment works with a population equivalent of 50.

### Field instrumentation

Our water quality monitoring programme was co-designed with a local rivers group (River Chess Association) who wanted to understand how a local wastewater treatment works was influencing water quality in the River Chess. Four Eureka Manta 2 water quality sondes were installed in the River Chess from April 2019 and programmed to take measurements at 15-minute intervals. Each sonde was equipped with sensors for measuring water temperature, pH, electrical conductivity, turbidity and dissolved oxygen. The temperature-compensated electrical conductivity (reported as corrected to 25 $\rm{^{o}C}$) and water temperature datasets are the focus here. The sondes were fitted with an extended wiper arm to clean the sensors prior to each measurement, and were manually cleaned and checked every two weeks by our Citizen Scientist team to prevent fouling. The calibration for each sensor was checked monthly by the academic team. Water temperature and electrical conductivity remained within the detection range of the sensors throughout the study (– 5 to 50$\rm{^{o}C}$ for water temperature and 0 to 100 mS/cm for electrical conductivity).

The Citizen Science programme was designed to investigate water quality downstream of the urbanised headwaters of the River Chess. Accordingly Sensor 1 (BH) was installed downstream of Chesham (the town in the headwaters of the River Chess); Sensor 2 (LC) was installed in a side channel of the River Chess within the urbanised reaches which receives water from an artesian well and natural spring; Sensor 3 (LP) was installed approximately 2 km downstream of Chesham Wastewater Treatment Works (WWTW) but upstream of any other major tributaries or springs; and Sensor 4 (WB) was located 3 km downstream of Sensor 3 to indicate how far downstream effects from Chesham WWTW were detectable. There are numerous springs that contribute water to the River Chess between Sensor 2 and 3, see also Fig. 13 for a map.

Fifteen-minute rainfall total (Chenies, Station Number 278744TP), river discharge (Rickmansworth gauging station Number 2859TH) and groundwater level data (Ashley Green) were obtained from the Environment Agency. Chesham Wastewater Treatment Works treated effluent discharge data (15-minute resolution) was obtained from Thames Water^46^. Note that we use “treated effluent discharge” to measure the amount of final, treated effluent released from the plant.

### Data analysis

Data analysis was carried out on 12 months of 15-mins sensor data (1 June 2019 to 1 June 2020). Empirical probability density functions (PDF) were derived for electrical conductivity and temperature in order to compare summary statistical properties associated with the four monitoring sites. Dominant frequencies in the electrical conductivity and temperature datasets were identified using Fourier analysis and compared to cyclic patterns in the WWTW treated effluent discharge in order to test whether electrical conductivity could act as a proxy for the dominance of WWTW discharge. GAM and gradient boosted tree analysis were applied to the 12-month dataset to investigate the influence of different variables and their interactions (time, water level, pH) on electrical conductivity. To run the analysis, we shuffled the data and split it into 70% training and 30% test data. Finally SHAP analysis was applied to the boosted tree outputs to aid detailed interpretation of the results. Each of the data analysis methods is described in detail in Sections a–f below. Note that all data and code is freely available online (see code availability statement). Hence, we encourage the interested reader to consult the code in addition to the general, more high-level descriptions offered here.

- * Empirical probability density functions (PDF)* To evaluate how likely a certain observation *y* is, we use empirical probability density functions (PDFs). Each measurement instance $y_1$, $y_2$, $y_3$, ... $y_j$, ..., $y_N$ is aggregated into one of *n* bins. Each bin *i* covers an interval $\left[ y_\text {min}(i),y_\text {max}(i)\right)$, where we have $y_\text {min}(i+1)=y_\text {max}(i)$. After counting how many measurement fall into each bin *i*, we normalize by the total number of counts so that each bin represents a probability *p*(*i*) with $\sum _i p(i)=1$. Thereby, we obtain a normalized histogram. In addition to this histogram, we also display an empirically fitted curve, which is the univariate kernel density estimate, i.e. it is a function approximating the underlying histogram as
  1 $$\begin{aligned} p(y)\approx \frac{1}{n} \sum _j K(y-y_j), \end{aligned}$$
  where we chose a Gaussian kernel *K*. Technically, we estimate and display the empirical densities using the *seaborn* package in Python^68^.
- *Fourier analysis* In many ecological systems, we observe periodicity, e.g. in terms of seasonal or daily cycles. To analyse these cycles, we employ Fourier analysis, which transforms a time series *y*(*t*) from the time domain, i.e. using the argument *t*, to the frequency domain:
  2 $$\begin{aligned} {\tilde{y}}(k)=\int _{-\infty } ^{\infty } y(t)\cdot \text {e}^{-i2\pi kt}\text {d}t, \end{aligned}$$
  where *i* is the imaginary unit. The new series ${\tilde{y}}(k)$ is a function of frequencies *k* and we apply an inverse Fourier-transform to re-obtain the original time series *y*(*t*). Cyclic behaviour is easy to analyse using ${\tilde{y}}(k)$, as ${\tilde{y}}(k)$ will peak at intrinsic frequencies of the time series *y*(*t*). For example, a time series which is exactly a sine function with period 1 hour will lead to a delta function of ${\tilde{y}}(k)$ at $k=(1h)^{-1}$, while a realistic time series with several frequencies and a pronounced daily cycle will display a finite peak at ${\tilde{y}}\left( (24h)^{-1}\right)$. The larger the peak in the Fourier transform ${\tilde{y}}(k)$, the more dominant is this frequency in the original time series *y*(*t*).
- * GAM* As one possible approach to derive how the different variables (features) impact our target, we employ Generalized Additive Models (GAMs)^31^. GAMs use splines, i.e. piece-wise smoothly connected polynomials, to describe local dependencies. Several of these splines are added to obtain a complete model of the relationship between the different state space quantities. In particular, to describe the observable *y* we build the following model:
  3 $$\begin{aligned} y=c+s_{1}(x_{1})+s_{2}(x_{2})+..., \end{aligned}$$
  where *c* is a constant (intercept or bias) and $s_{i}$ are 3rd order B-splines for each of the features $x_{i}$ and we simply add all spline terms together, leading to an additive model. Technically, we implement GAMs by utilizing the Python pyGAM package^69^ and use a 70% training and 30% test split of randomly shuffled data. For consistency, we use an identical number of splines at all sites, namely 10. A slightly lower error can be achieved by fine-tuning the number of splines to each site, approximately reducing the error by up to $5\%$.
  An upside of GAM is its straightforward interpretability. No further steps are necessary to obtain partial dependencies from a GAM approach, we can simply visualize the splines $s_{i}$ to see how a given feature $x_i$ influences our target *y*.
- * Gradient boosted trees* As an alternative to GAMs, we also employ gradient boosted trees to describe the feature interaction and inter-dependencies. The key idea is that an ensemble of “weak learners”, such as unbiased but high-variance trees, is used to generate one much more precise, ideally low-bias and low-variance predictor. We initialize a single tree, then compute the loss, i.e. the error in its prediction on a validation set, and compute the gradient of this loss. Next, we fit a new regression tree on the gradients. The new predictor is obtained by summing the newly fitted tree with the previous predictor. A learning rate $\eta$ controls how much we move along the gradient and thereby how much the next added tree modifies the previous prediction. For this updated predictor, consisting of a sum of trees, we again compute the loss, the gradient and perform an update. This process is repeated until a certain number of iterations has been completed or a loss threshold has been passed.
  On the technical side, Boosted is implemented in Python utilizing LightGBM^70^ and we find suitable hyperparameters using FLAML^58^, i.e. an automated machine learning framework that explores possible parameters automatically. We restricted the hyperparameter exploration to 1000 seconds and achieved high precision. Some testing revealed that the interpretation of the models is only slightly influenced by changing the time allocated to the parameter search, e.g. down to 100 seconds. Hence, we are confident that the results obtained and discussed below are independent of the specific hyperparameter solutions employed. We allow hyperparameters to vary for each model and obtain learning rates of the order $\alpha \sim 0.02...0.05$ and number of leaves $\sim 300$. Details on the implementation are available in the published code.
- * Shapley values* In contrast to GAMs, boosted trees require more effort to allow for a detailed interpretation. Here, we interpret the fully trained tree by applying Shapley values^57^. The idea of Shapley values originates from game theory, where it quantifies how much each player of a cooperative game contributed to the gained value. Hence, a winning coalition of players could split the rewards for winning a game fairly among its players by paying each player proportional to how valuable they were for the success.
  In machine learning, Shapley values answer a very similar question: Given a prediction outcome of a machine learning model (in our case boosted trees): How much did each feature (instead of a player) contribute to the decision reached by the model? More precisely, the Shapley value of feature *i* is the impact of the feature weighted and summed over all possible feature combinations:
  4 $$\begin{aligned} \phi _i(val)=\sum _{S\subseteq {x_1,...,x_p}\backslash {x_i}}\frac{|S|!(p-|S|-1)!}{p!}\left( val(S\cup {x_i}) -val(S) \right) , \end{aligned}$$
  where *S* is the set of features used in the model and $x=(x_1, x_2, ..., x_p)$ is the feature vector of the instance that should be explained. Furthermore, *p* is the number of features and *val*(*S*) is the prediction for the target *y* given the features in the set *S*. Now, we can evaluate the contribution in different feature coalitions by varying which features enter in *S* and which are marginalized. Critically, Shapley values have several desirable properties, such as being efficient, symmetric, additive and invariant under the addition of a dummy feature, see also^57^ for more details.
  While Shapley values are often computationally very demanding to compute,^36^ and^37^ introduced SHAP (SHapley Additive exPlanations), alongside computationally efficient algorithms to compute SHAP values for tree-based methods. Similar to Shapley values, SHAP tells us how much each feature contributes to a prediction. Specifically, a positive SHAP value tells us that a given feature will push the prediction above the mean value, while a negative SHAP value means the feature typically reduces the predicted value. The magnitude of the SHAP value can then be used to rank features (feature importance). Finally, partial dependency plots are obtained by plotting the feature value vs its SHAP contribution, i.e. plotting pairs $(\phi (x_i),\phi _i)$ for a given feature *i*.
- *Data cleaning* When applying either the GAM or the boosted tree approach, we rely on clean data sets without any gaps or NaN (not a number) entries. Hence, when preparing the data set for the training-test split, we eliminate each row where at least one entry is missing or NaN. While this reduces the available data, we avoid imposing any modelling assumptions necessary to impute the missing data. Note that not all measurement sites have NaN at the same time and as a consequence, we might only be able to model the summer and autumn at one site, while modelling the whole year at another site, leading to different ranges of the “month” value. The total number of “clean” data points left for the different sites is about 17000 for LC and BH, 27000 for LP and 18000 for WB, corresponding to something between 177 to 288 total days of clean data. Most of this usable data is in a large, continuous time period.
  Finally, for the LC site, we noticed a systematic offset in electrical conductivity to lower-than-usual values for a short period which was due to an obstruction in the sensor cavity. We corrected this offset by increasing the values to match the following time period, see published code for details.

## Supplementary Information

Supplementary Information.

## Data Availability

Data from the river Chess is available on the following ChessWatch website https://rhysh.shinyapps.io/ChessWatch/. The code that was used to analyse the data is available at https://osf.io/txjv3/.
