# Supplementary material for: Machine learning approach towards explaining water quality dynamics in an urbanised river
Source: Sci Rep. 2022 Jul 19;12:12346. doi: 10.1038/s41598-022-16342-9 (PMC9295889; doi:10.1038/s41598-022-16342-9)
Supplement: Supplementary file 1 — Supplementary Information. [file 41598_2022_16342_MOESM1_ESM.pdf]

Supplementary Information for "Machine learning approach towards explaining water quality dynamics in an urbanised river"

Benjamin Schäfer,[1,2,3,*] Christian Beck,[1,4] Hefin Rhys,[5] Helena Soteriou,[6] Paul Jennings,[7] Allen Beechey,[8] and Catherine M. Heppell[9]

[1] *Queen Mary University of London, School of Mathematical Sciences, Mile End Road, London E1 4NS, UK*
[2] *Faculty of Science and Technology, Norwegian University of Life Sciences, 1432 Ås, Norway*
[3] *Institute for Automation and Applied Informatics, Karlsruhe Institute of Technology, 76344 Eggenstein-Leopoldshafen, Germany*
[4] *The Alan Turing Institute, 96 Euston Road, London NW1 2DB, UK*
[5] *The Francis Crick Institute, Flow Cytometry Science Technology Platform, London, UK*
[6] *Thames Water, Clearwater Court, Vastern Road, Reading RG1 8DB, UK*
[7] *River Chess Association, UK*
[8] *Chilterns Chalk Streams Project, Chilterns Conservation Board, Chinnor, Oxfordshire, OX39 4HA,UK*
[9] *Queen Mary University of London, School of Geography, Mile End Road, London E1 4NS, UK*

Within this Supplementary Information, we provide complementary analysis for the results shown in the main manuscript. In particular, we show results of GAM and boosted tree analysis for the locations not shown in the main text. Furthermore, we highlight how we estimate the time delays when aligning the different time series.

* corresponding author: benjamin.schaefer@kit.edu

Supplementary Note 1.

We present the GAM analysis for the two upstream measurement sites BH and LC in Figure 1. GAM results of WB and LP are reported in the main text.

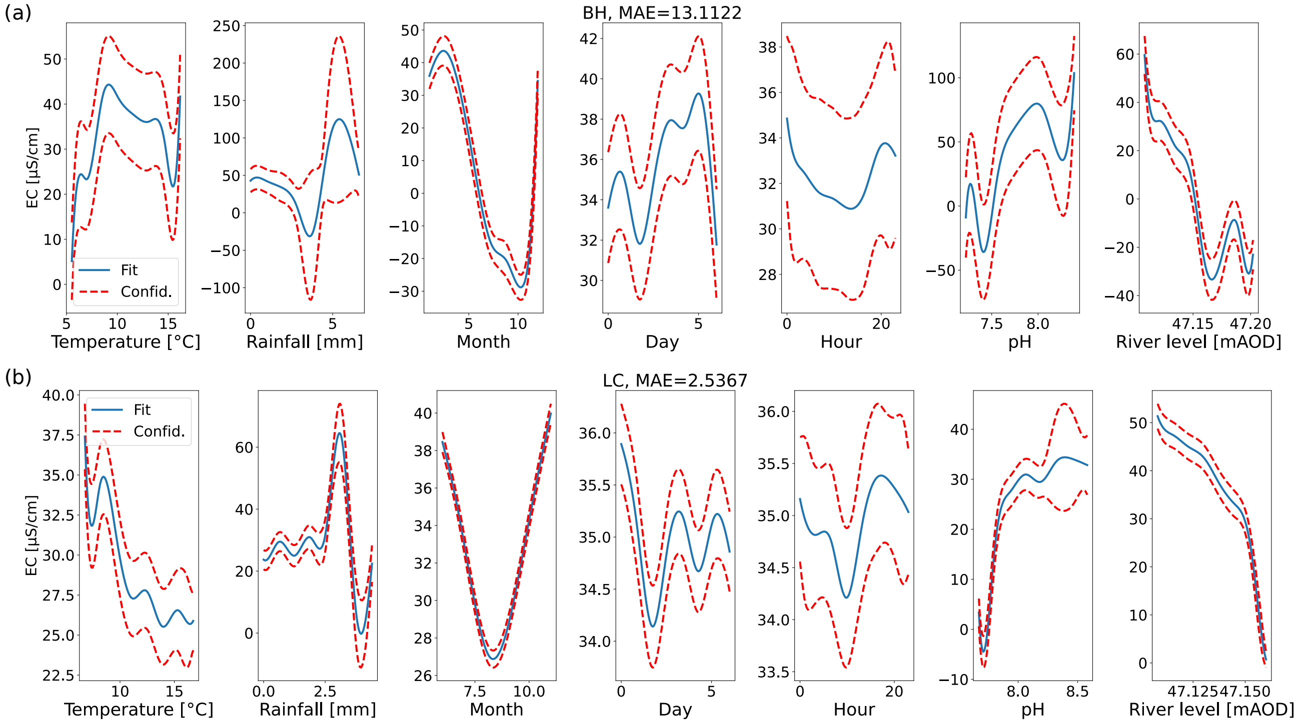

Supplementary Figure 1. Results of the GAM analysis for BH (a) and LC (b). Best fitting splines of the different features x_i and their influence on the electrical conductivity (EC) in the fully-fitted GAM approach. The blue curve gives the best fit and the red dashed lines envelop a single confidence interval (68% assuming an underlying Gaussian uncertainty). Finally, we report the mean absolute error (MAE) when the model is applied to the previously withhold test set.

Supplementary Note 2

We present the boosted tree for the two upstream measurement sites BH and LC in Figure 2 and 3. Boosted tree results of WB and LP are reported in the main text.

From Figures 2 and 3, we observe that the two upstream locations do not depend as much on the river level, which was the most important feature for the two downstream sites.

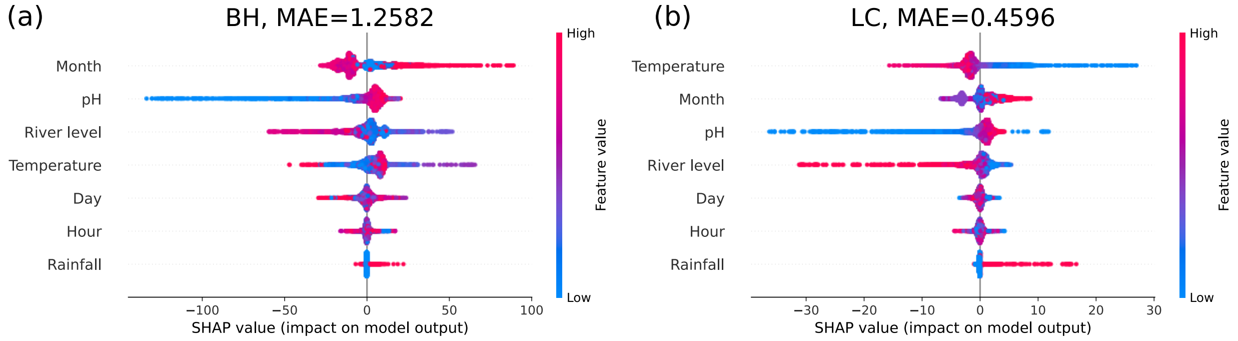

Supplementary Figure 2. Feature importance of the boosted tree approach for BH and LC.

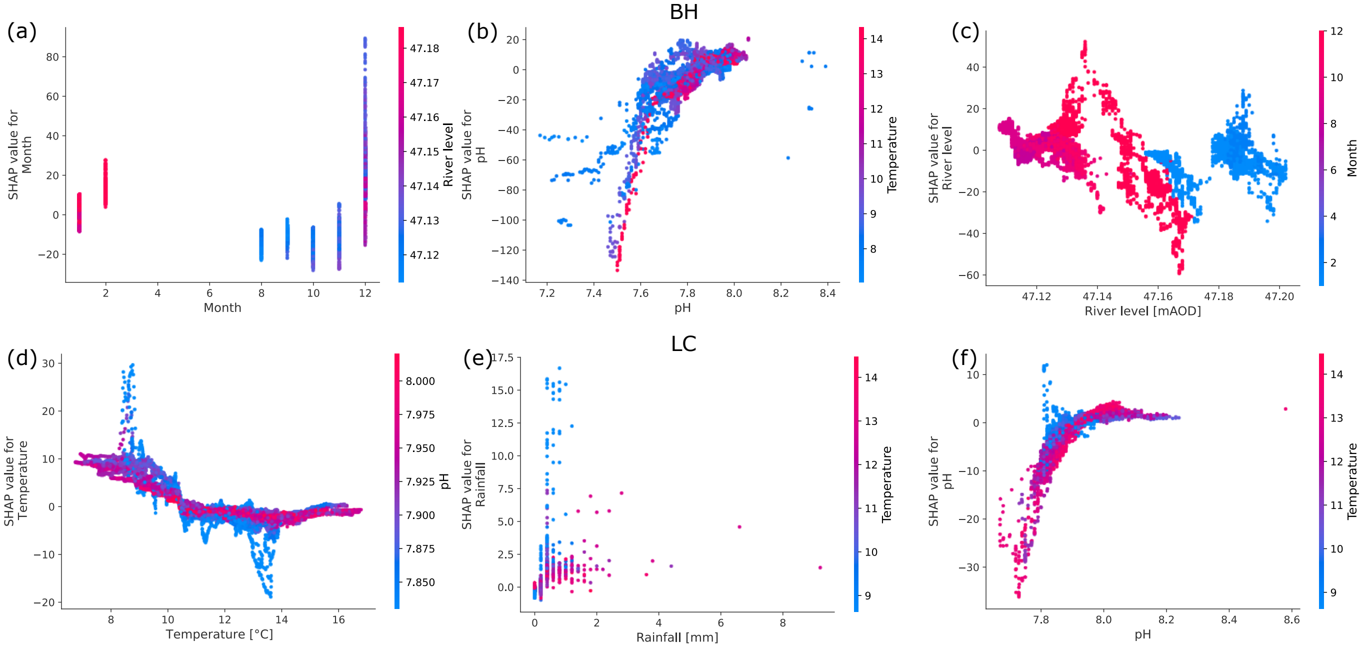

Supplementary Figure 3. Partial dependency plot of the three most important features at the BH (a) and LC (b) measurement sites and their influence on the electrical conductivity (EC) in the fully-fitted boosted tree approach.

Supplementary Note 3

In the main text we have discussed that the WWTW discharge is highly correlated with the EC measurements at the LP and WB sites once an appropriate time shift is included. Based on our measurements, we estimated that it takes 2.5 hours from the WWTW at Chesham to LP and another 6 hours from LP to WB. Finally, we utilize total river flow measurements (to compute the fraction of how much the WWTW discharge contributes to the total flow) at Rickmansworth [1], which lies even further downriver. Based on the recorded river level at Chesham and Rickmansworth, we estimate the time delay as between these two points as 15.25 hours. This leads to a time delay of approximately 6.75 hours from WB to Rickmansworth, see also 4.

Note that we only align the flows, i.e. the total river flow, the WWTW discharge and the WWTW fraction, while we utilize the original time stamps for the river level as this is mostly driven by rain events affecting all parts of the river at the same time.

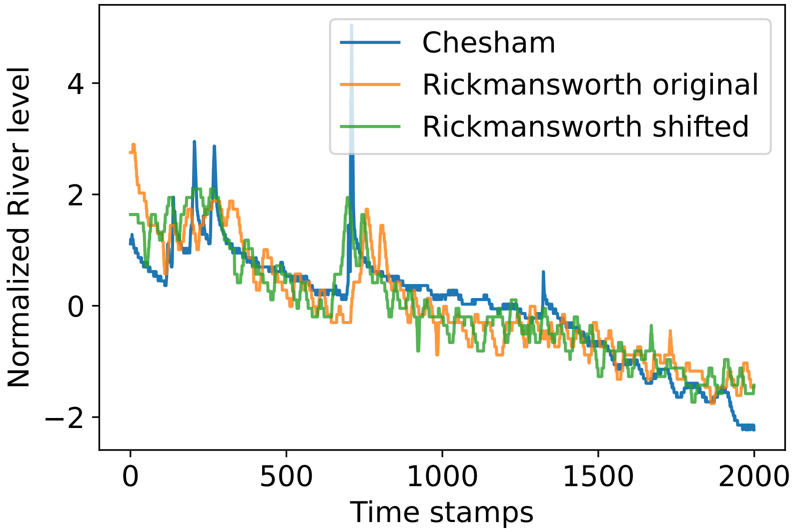

Supplementary Figure 4. Normalized river levels recorded at Chesham and Rickmansworth align well once a shift of approximately 15.25 hours is included. Note that the river Chess is a chalk stream and therefore exchanges considerable amounts of water with the groundwater reservoir. Hence, river levels do not simply propagate from one location to the other.

Supplementary References

[1] NERC Centre for Ecology and Hydrology. National river flow archive 2020: National river flow archive. http://nrfa.ceh.ac.uk (2020). (accessed 27 October 2020).
